# Identification and Phylogeny of the First T Cell Epitope Identified from a Human Gut *Bacteroides* Species

**DOI:** 10.1371/journal.pone.0144382

**Published:** 2015-12-04

**Authors:** Maria Elisa Perez-Muñoz, Payal Joglekar, Yi-Ji Shen, Kuan Y. Chang, Daniel A. Peterson

**Affiliations:** 1 Department of Pathology, Johns Hopkins University School of Medicine, Baltimore, Maryland, United States of America; 2 Department of Microbiology and Immunology, Stanford University School of Medicine, Stanford, California, United States of America; 3 Department of Food Science and Technology, University of Nebraska, Lincoln, Nebraska, United States of America; 4 Department of Computer Science and Engineering, National Taiwan Ocean University, Keelung, Taiwan; INSERM, FRANCE

## Abstract

Host T cell reactivity toward gut bacterial epitopes has been recognized as part of disease pathogenesis. However, the specificity of T cells that recognize this vast number of epitopes has not yet been well described. After colonizing a C57BL/6J germ-free mouse with the human gut symbiotic bacteria *Bacteroides thetaiotaomicron*, we isolated a T cell that recognized these bacteria *in vitro*. Using this T cell, we mapped the first known non-carbohydrate T cell epitope within the phylum *Bacteroidetes*. The T cell also reacted to two other additional *Bacteroides* species. We identified the peptide that stimulated the T cell by using a genetic approach. Genomic data from the epitope-positive and epitope-negative bacteria explain the cross-reactivity of the T cell to multiple species. This epitope degeneracy should shape our understanding of the T cell repertoire stimulated by the complex microbiome residing in the gastrointestinal tract in both healthy and disease states.

## Introduction

The specificity of T cells that recognize epitopes in the gut microbiota has not yet been well described, likely due to the vast number of epitopes involved and the complexity of host-gut microbiota interactions. However, a few enlightening studies have been reported. In inflammatory bowel diseases (IBD), Piezer and colleagues [1] showed that the disease-specific intestinal lesions were caused by T cell reactivity towards bacterial antigens. Subsequently, Duchmann *et al*. [2] showed that individual T cell clones from IBD patients were reactive to *Bacteroides thetaiotaomicron*, *Bifidobacterium bifidum*, *Escherichia coli*, and *Yersinia enterocolitica*. In 2008, Duchmann and colleagues [3] expanded their studies on antigenic epitopes by screening T helper cells from healthy subjects and from patients with Crohn’s disease and ankylosing spondylitis. However, none of these studies identified the actual epitope that stimulate the T cell. This is an important step to clarify the interaction between host and bacteria on a molecular level.

In order to study this issue, we developed a novel approach to identify these epitopes. We used gnotobiotic mice and bioinformatics to examine the complex patterns of shared epitopes within the genus *Bacteroides*. We focused on *Bacteroides* because it represents a major component of the human gut microbiota [4] and studies have found gut *Bacteroides* altered in a wide range of diseases. Increases in *Bacteroides* communities have been found in IBD, gastritis and arthritis [5,6] and have also been associated with various other pathologies, including colorectal cancer, diabetes, and obesity [7–10]. The presence of *Bacteroides* spp. early in life may be an early indicator of asthma development later in life [11–12]. Finally, *B*. *ovatus*, *B*. *vulgatus*, and *B*. *thetaiotaomicron* have been specifically implicated in the development of IBD and celiac disease [4,13–15].

As a result of our studies, we identified the first peptide T cell epitope within the phylum *Bacteroidetes*. We also found that this T cell responded to multiple species of bacteria. By using two powerful tools—gnotobiotic mice and microbial genetics—we mapped the antigenic peptide of the T cell primed by *B*. *thetaiotaomicron in vivo*. Also, by using a genomics/bioinformatics based analysis, we discovered that we could predict the pattern of species recognition of these T cells based on the frequency of shared peptides within the genus *Bacteroides*.

## Materials and Methods

### Bacterial strains

*Bacteroides* species were grown in Tryptone Yeast Glucose broth (TYG) [16] in an anaerobic chamber (37°C, 5% CO_2_, 5% H_2_ and 90% N_2_) and included *B*. *caccae* ATCC 43185^T^, *B*. *dorei* JCM 13471, *B*. *eggerthii* ATCC 27754, *B*. *fragilis* NCTC 9343, *B*. *finegoldii* JCM13345, *B*. *intestinalis* JCM 13265, *P*. *merdae* ATCC 43184, *B*. *ovatus* JCM 5824, *B*. *sartorii A-C2-0*, *B*. *stercoris* ATCC 43183, *B*. *thetaiotaomicron* VPI 5482, *B*. *uniformis* JCM 5828^T^, and *B*. *vulgatus* ATCC 8482. Non-*Bacteroides* species included *Prevotella stercorea* JCM 13469^T^ grown using Peptone Yeast Glucose broth (PYG, DSMZ Medium 104) and *Escherichia coli* grown in Luria Bertani broth (LB, BD 244620).

### T cell hybridomas and antigen presenting cells (splenocytes)

The 52.13 hybridomas were created using previously established protocols [17] by stimulating splenocytes from an ex-germ-free C57BL/6J mouse colonized with *B*. *thetaiotaomicron* VPI 5482 [17]. Hybridomas were maintained in a tissue culture incubator (5% CO_2_, 37°C) in DMEM (Hyclone sh30003.03) modified as follows: 116mg/l of L-arginine, 36mg/l of L-asparagine, 2g/l of NaHCO_3_, 1mM sodium pyruvate, 1.5mM L-glutamine, 10mM HEPES, 100units/l of Pen/Strep, and 5 x10^-5^M of beta-mercaptoethanol. Colonization was performed by single gavage of 1 x 10^8^ bacterial cells grown in TYG broth. The spleen of the *B*. *thetaiotaomicron*-colonized mouse, which received autoclaved water and autoclaved standard chow ad libitum, was harvested 14 days post-colonization. Splenic T cells were stimulated *in vitro* with heat killed *B*. *thetaiotaomicron* (HKBT) for 3 days and then fused to the BW5147 to create T cell hybridomas. All spleen-donor mice (the axenic mouse for the creation of the hybridomas and the conventional mice for T cells assays described below) were euthanized inside a biosafety cabinet using the Isoflurane drop-jar method followed by cervical dislocation. Mice were handled according to approved protocols established by the Animal Care and Use Committees at the University of Nebraska and Johns Hopkins University (A3459-01 and M014M345). All efforts were made to minimize animal suffering. Refer to S1 Appendix for compliance of the ARRIVE guidelines.

### Assay for screening reactivity to *Bacteroides* species and transposon mutants

T cell hybridoma cells (5 x 10^4^) were mixed per well with freshly harvested mitomycin c-treated splenocytes (5 x 10^5^) from conventional C57BL/6J mice. The donor mice were housed in ventilated racks and received water and standard rodent chow ad libitum. One spleen was used for 2 to 3 assays (performed in 96 wells plates), depending on the number of harvested splenocytes, to keep animal use to a minimum. The hybridoma was stimulated with 5μl of bacterial antigen. After 24 hours of incubation at tissue culture conditions (37°C, 5% CO2), supernatants were transferred into sterile 96 wells plates and IL-2 secretion was detected by CTLL-2 bioassay with ^3^H-thymidine incorporation. CTLL-2 cells (2 x 10^4^) in 100μl volume were added per well. Plates were incubated for additional 22 to 24 hours followed by the addition of 20μl of tritiated thymidine (^3^Ht, 0.4μCi/well). After a last incubation period of 22–24 hours, cells were harvested (Inotech IH-110-96S) into glass fiber filters (Perkin Elmer 1450–421), and ^3^Ht incorporation was measured in a 1450 Micro Beta™ liquid scintillation counter. We looked for positive proliferation in wells stimulated with the wild type (representative) bacterial strain and for negative proliferation when screening transposon mutants, as mutants that failed to stimulate the hybridoma did not contain the epitope. All assays were performed at least twice with replicates in duplicates or triplicates. Statistical significance was determined by one-way analysis of variance using Graph Pad Prism version 4.0b.

### Transposon library and sequencing

In order to identify the epitope recognized by the hybridoma, a mutant library of *B*. *thetaiotaomicron* was created using previously established protocols for transposon mutagenesis [18]. The site of transposon insertion in the negative *B*. *thetaiotaomicron* mutants was determined using arbitrary nested PCR (AP-PCR) with several sets of primers. In AP-PCR, primers that bind regions of known sequence (such as the transposon) are paired with primers of arbitrary sequence in a first round of PCR. The arbitrary primers bind multiple sites in the genome including the known region where the transposon randomly inserts. The product of the first PCR is used as template in a second nested PCR round using sequence specific primers paired with one of the arbitrary primers used in the first round.

DNA was extracted from the bacterial cultures using the DNeasy Blood and Tissue Kit (QIAGEN #69506) following manufacturer’s instructions. The SAMseq primers published by Goodman and colleagues [19] were used for two rounds of PCR and sequencing of the final product (performed at the Johns Hopkins Sequencing Center). In addition, AR1B (5’-GGCCACGCGTCGACTAGTACNNNNNNNNNNGATGC-3’) and AR2 (5’-GGCCACGCGTCGACTAGTAC-3’) were used in round one and two, respectively. PCR reactions consisted of 5μl of 10X Taq buffer, 3μl of 50 mM MgCl2, 1μl of 10mM dNTPs, 1.25μl of the primers at 20μM, 0.4μl of taq polymerase and 1μl of bacterial DNA (for the first PCR round), or 2μl of the PCR product (for the second round), and nuclease-free water to complete 50ul per reaction tube. A BioRad T100 Thermal Cycler was used for amplification as follows: 2 minutes at 95°C; followed by 5 cycles of 1 minute at 95°C, 1 minute at 30°C, and 1 minute at 72°C; followed by 30 cycles of 1 minute at 95°C, 1 minute at 55°C, and 1 minute at 72°C, and a final step of 1 minute at 72°C for round one. The second round consisted of: 2 minutes at 95°C; followed by 40 cycles of 1 minute at 95°C, 1 minute at 55°C, and 1 minute at 72°C; and lastly 2 minutes at 72°C. Amplicons from individual samples were purified using the MiniElute PCR Purification Kit (QIAGEN #28006). Reads obtained from sequencing were mapped to the *B*. *thetaiotaomicron* VPI 5482 genome sequence using the NCBI nucleotide BLAST function for determination of transposon insertion.

### Expression of BT0900

The BT0900 gene from *B*. *thetaiotaomicron* VPI 5482 genomic DNA was PCR amplified using AccuPrime™ Pfx DNA Polymerase (Invitrogen) and gggaattccatatgatgaagaatgtaaaagaagccaaaa and aaaaggatccttatttcatcatcttatcgatttcttca as forward and reverse primers, respectively. Amplification conditions were 95°C for 15 seconds, 50°C for 30 seconds, 68°C for 1.5 minutes for 25 cycles. The gene was cloned into the pET15b vector (Novagen), followed by transformation into BL21(DE3)pLysS competent cells for protein expression. BL21(DE3)pLysS cells carrying pET15b[bt0900] were grown in LB medium containing ampicillin (100μg/ml) to an OD of 0.6. Isopropyl-1-thiogalactopyranoside (IPTG) was added to a final concentration of 1.0 mM to induce the recombinant protein expression for 3 hours. At this stage the cells were either heat inactivated at 80°C for 1 hour to be used for T cell hybridoma assay, or subjected to protein purification in order to obtain pure His-tagged Bt0900 recombinant protein. For purification, the cells were resuspended in a buffer containing 50 mM NaH_2_PO4 (pH 8.0), 300 mM NaCl, 10 mM imidazole, and 10 Units Dnase and lysozyme (1mg/ml). Subsequently, the cells were incubated on ice for 30 min, followed by sonication (10s pulse followed by 5s pause for 6 times), and lastly, centrifuged to collect supernatant for protein purification. The supernatant was added to a pre-equilibrated column of Ni^2+^–NTA agarose (Qiagen), washed with 100 ml of 50 mM NaH_2_PO4 (pH 8.0), 300 mM NaCl, 10 mM imidazole; and eluted in five 1 ml fractions with 50 mM NaH_2_PO4 (pH 8.0), 300 mM NaCl, 250 mM imidazole. Protein concentration was achieved using Amicon® Ultra-15 Centrifugal Filters.

### Epitope mapping using synthetic peptides

Thirty-nine synthetic peptides (GenScript), consisting of 15 amino acids (aa)—overlapping each other by 5 aa—were designed based on the BT0900 gene aa sequence, and tested at 100μM, 10μM and 1μM concentrations for reactivity in a T cell-CTLL assay as described before. Upon selection of the positive peptide (peptide 36), six new 12-aa long peptides (based on the sequence of the previously identified positive peptide and overlapping each other by 11aa) were designed (Peptide 2.0) and screened with the purpose of identifying the epitope recognized by 52.13.

### Assay with neutralizing MHCII antibody to block epitope presentation

A T cell hybridoma assay was performed to demonstrate blocking of 52.13 activation in the presence of its epitope. After harvesting and preparing cell suspension from spleen, splenocytes (5 x 10^5^/well) were treated with 4-fold serial dilutions of purified anti-mouse I-A/I-E antibody (Biolegend, clone M5/114.15.2) starting at 2000ng/ml in the presence of 10-fold serial dilutions of peptide 36 (from 100uM to 0.1uM), 0.5ul/well of *B*. *thetaiotaomicron* VIP 5482 or no antigen, and incubated for 30 minutes at 4°C. Subsequently, 52.13 hybridomas (5 x 10^4^) were added and the plate was incubated at tissue culture conditions for 22–24 hours. Assay was completed as described above for screening of reactivity to *Bacteroides* species and transposon mutants.

## Results

### Degenerate recognition of multiple species by a single T cell hybridoma

**Fig 1:**
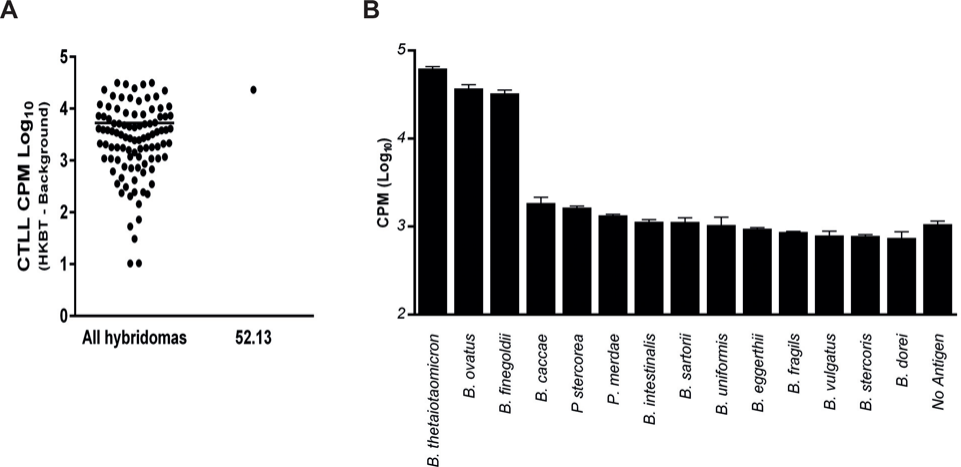
Peripheral T cells poly-recognize gut bacterial species. T cell hybridomas were generated from C57BL/6J mice monoassociated with *B*. *thetaiotaomicron* VPI 5482 after *in vitro* stimulation with heat-killed bacteria. A) Hybridomas were screened for IL-2 secretion, measured by proliferation of CTLL-2 bioindicator cells B) The 52.13 hybridoma showed reactivity to 3 species of bacteria. Lines above bars indicate standard deviations.

Hybridomas were created by fusing splenocytes from *B*. *thetaiotaomicron* monoassociated mice that were stimulated *in vitro* with HKBT. These hybridomas were then screened for IL-2 secretion by stimulation with APC and HKBT or a no antigen control (Fig 1A). It was striking that most of the tested hybridomas had significant IL-2 levels above background, suggesting that bacteria-reactive T cells are not rare. From among the hybridomas with the highest reactivity, 52.13 was chosen for further analysis because it had a very robust response and clone stability that maintained reactivity *in vitro*. The 52.13 T cell-CTLL assay showed strong responses (>10,000 CPM, P<0.0001) to *B*. *thetaiotaomicron*, *B*. *ovatus* and *B*. *finegoldii* (Fig 1B). However, there is no indication that this T cell (52.13) was a dominant or frequent member of the T cell repertoire.

### Determination of negative mutants: Screening bacterial transposon mutants is an effective tool for mapping T cell responses to bacterial antigens

**Fig 2:**
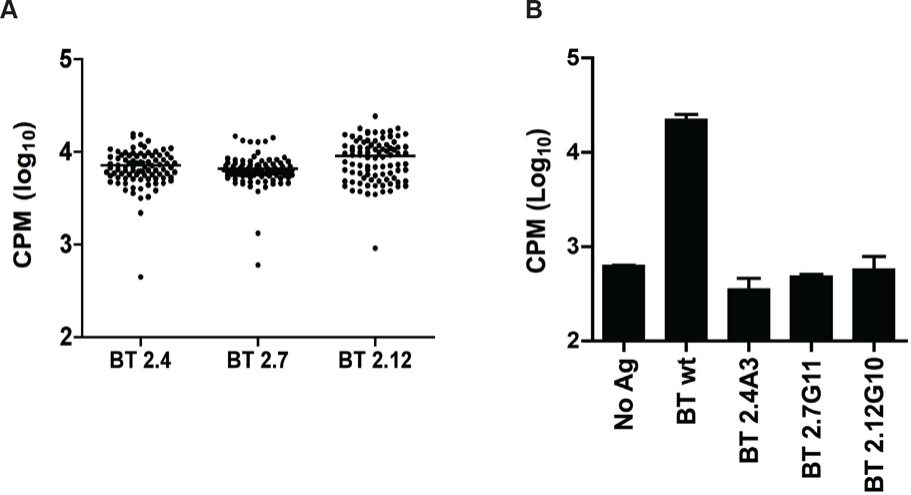
Screening transposon mutants is an effective tool for mapping T cell responses to bacterial antigens. A library of transposon mutants of *B*. *thetaiotaomicron* VPI 5482 arrayed in 96 well plates was screened to identify non-stimulatory mutants. A) The CTLL-CPM of mutant screening plates where negative mutant were first identified. B) Mutants identified in the screen were regrown from glycerol stocks and confirmed as negatives (n = 2). Lines above bars indicate standard deviations.

Unlike mapping peptides from a single protein, a peptide library of all the peptides of *B*. *thetaiotaomicron* was not feasible (there are about 1.8x10^6^ possible peptides). We used a method that we previously demonstrated effective for mapping IgA epitopes [18]. Screening of over 1,500 *B*. *thetaiotaomicron* transposon mutants resulted in the identification of three mutants (2.4A3, 2.7G11 and 2.12G10) that did not elicit IL-2 production by 52.13 (Fig 2A). Negative mutants were expanded from glycerol stocks, streaked to isolate single colonies, and retested to confirm loss of the epitope (Fig 2B).

### The *B*. *thetaiotaomicron* BT0900 protein activates the 52.13 CD4^+^ T cell hybridoma

**Fig 3:**
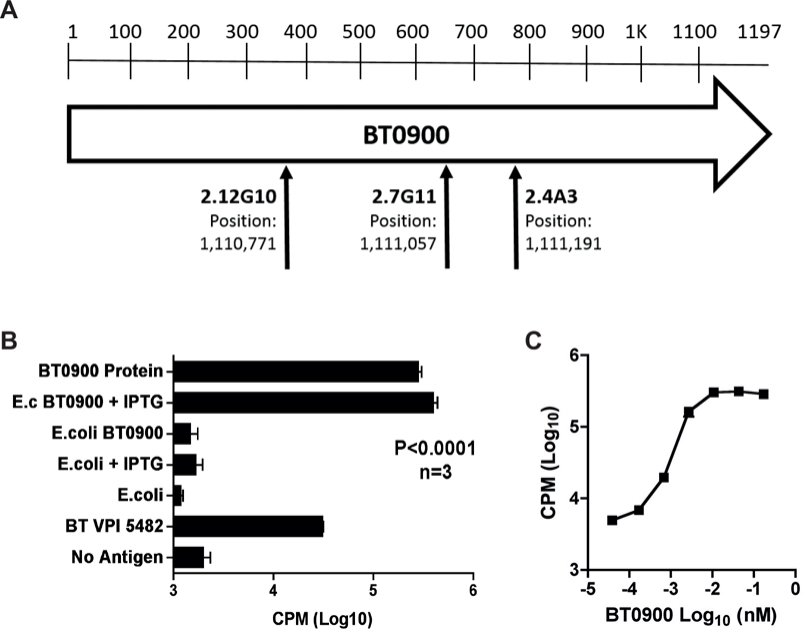
The *B*. *thetaiotaomicron* BT0900 protein activates the 52.13 CD4+ TCR. A) Sequencing the site of transposon insertion within the *B*. *thetaiotaomicron* VPI 5482 genome sequence demonstrated the 3 independent mutants all disrupted the BT0900 gene. This was observed by adding either *E*. *coli* with and without BT0900 expression induced with IPTG, or the histidine-tagged BT0900 purified from the bacteria over a nickel column. B) The expression of BT0900 in *E*. *coli* allowed the confirmation of this gene as encoding the protein containing the epitope for 52.13. Lines above bars indicate standard deviations. C) Titration of purified protein demonstrated the sensitivity of the T cell hybridoma.

Arbitrary-PCR was used to amplify the junction where the transposon was inserted into the *B*. *thetaiotaomicron* VPI 5482 genome [19]. We observed that the transposon insertion sites for mutants 2.4A3, 2.7G11 and 2.12G10 all disrupted gene BT0900 (Fig 3A). To confirm this result, BT0900 was expressed in *E*. *coli* and demonstrated to activate the 52.13 hybridoma (P<0.0001, Fig 3B). Purified BT0900 titration demonstrated that the T cell could respond to a picomolar range of antigen (Fig 3C).

### Peptide AKPFYEKARALK is the epitope of the 52.13 T cell hybridoma

**Fig 4:**
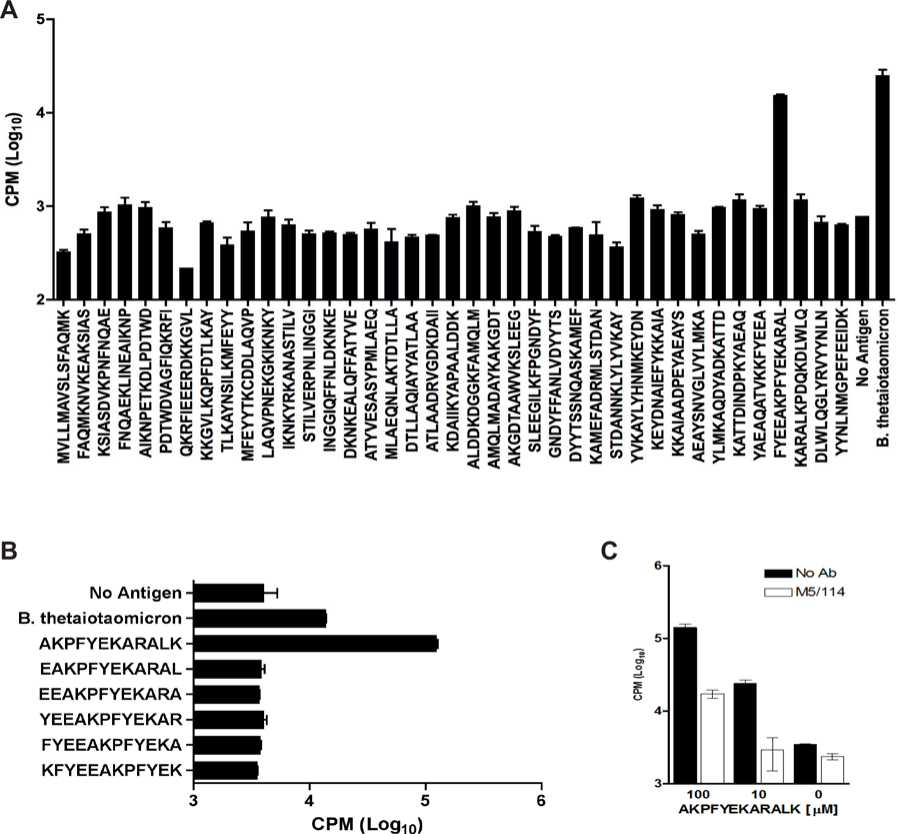
AKPFYEKARALK is the epitope for recognition of *Bacteroides* spp. by the 52.13 T cell hybridoma A) Screening of 39 synthetic 15 amino acid peptides demonstrated a strong response to the peptide in position 363 to 377 (FYEEAKPFYEKARAL) of BT0900. B) Screening of 12-mer peptides showed the peptide AKPFYEKARALK as the peptide epitope of the 52.13 T cell hybridoma. For graphs A and B, n = 2 and lines above bars indicate standard deviations. C). Addition of anti-mouse I-A (M5/114) antibody at 2000ng/ml blocks 52.13 reactivity to different concentrations of the epitope peptide AKPFYEKARALK.

The 52.13 T cell hybridoma was screened with a 15-mer overlapping library of synthetic peptides. The peptide FYEEAKPFYEKARAL is located in position 363–377 of the BT0900 sequence and strongly activated the 52.13 TCR (Fig 4A). Smaller overlapping peptides (12-mers) were designed and screened revealing AKPFYEKARALK as the 52.13 epitope (Fig 4B). Addition of anti-mouse I-A antibody blocks 52.13 reactivity to the epitope peptide (Fig 4C).

**Fig 5:**
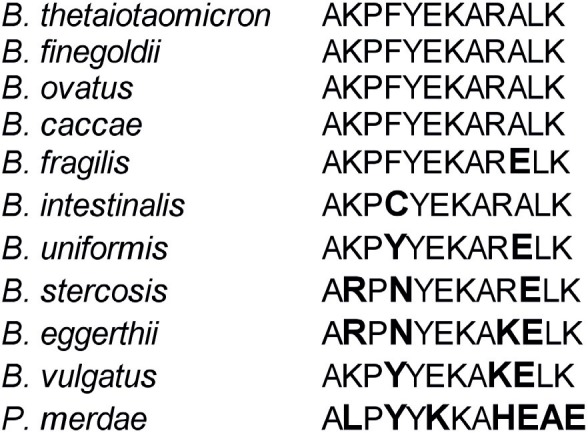
Peptide AKPFYEKARALK is common to *B*. *thetaiotaomicron*, *B*. *ovatus*, *B finegoldii* and *B*. *caccae*. Amino acid substitutions (bold) are seen in all other tested species that encode the protein, but do not stimulate 52.13.

Peptide AKPFYEKARALK is predicted to be present in the 3 positive species based on the sequence of their genomes, while the negative species had predicted polymorphisms within the peptide (Fig 5). The absence of stimulation by *B*. *caccae* could be the result of a lack of expression of the protein in this species when grown *in vitro*, or an error in the genome sequence.

### Understanding the patterns of shared peptides in gut *Bacteroides*

**Fig 6:**
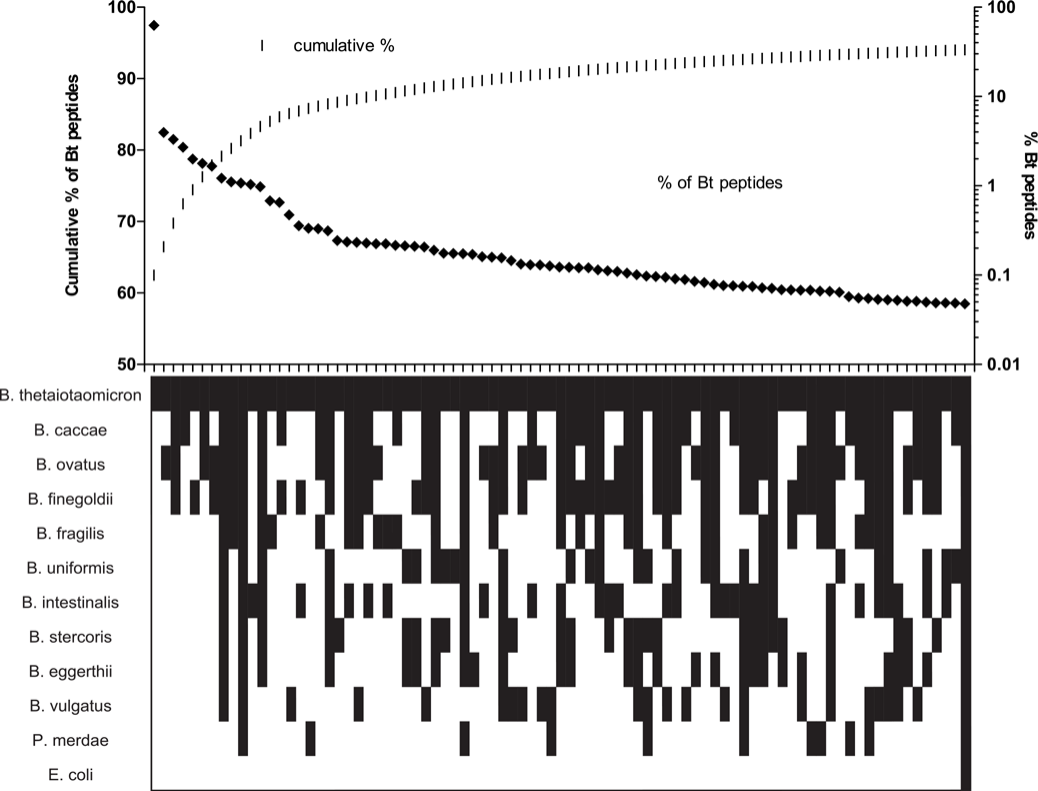
Prediction analysis of 12-mer peptides from 12 genomes. Each peptide was characterized based on the distribution of the 12-mer in each of the genomes (11 *Bacteroides* and one *E*. *coli* genome) with combinations from present in only one genome, to present in all genomes and every potential combination (total of 4096 possible combinations). Upper panel displays the percent of *B*. *thetaiotaomicron* peptides that fall into the most common patterns. ~62.5% of peptides of *B*. *thetatiotaomicron* are unique, the second most frequent pattern (~4%) is shared between *B*. *thetaiotaomicron* and *B*. *ovatus*. The left axis demonstrates the cumulative percent of the peptides that are described by the 85 most frequent patterns. The patterns of presence (black) or absence (white) are displayed for each pattern. Note that the 85^th^ most frequent pattern out of 4096 total patterns includes peptides shared between all genomes including *E*. *coli*, which is in the distant phylum *Proteobacteria*.

The 52.13 T cell was chosen from the repertoire due to its high sensitivity *in vitro*. This does not indicate and we do not suggest that it is a prevalent T cell in the repertoire of cells responding to *B*. *thetaiotaomicron in vivo*. To predict the frequency of shared peptides between related species of bacteria, we used an *in silico* approach as follows. Using the published genomic sequences of all the bacteria tested in our T cell assays, we analyzed all predicted 12-mer peptides. We then determined the pattern of shared peptides between the various genomes. This is a factorial process, where any peptide could be present in a single genome, all 12 genomes, or any combination of present or absence. Due to evolutionary history, a higher number of gene, protein and peptide sequences will be shared between bacteria the closer they are related. Therefore, we can predict that the most common patterns for a random 12-mer peptide coming from *B*. *thetaiotaomicron* will be 1) the peptide is unique to *B*. *thetaiotaomicron* is the most common, 2) the peptide will be found in *B*. *thetaiotaomicron* and *B*. *ovatus* is second most common, and 3) the third most frequent pattern will be peptides shared by *B*. *thetaiotaomicron*, *B*. *caccae*, *B*. *ovatus* and *B*. *finegoldii*. The pattern we observe for the 52.13 epitope is the third most frequent pattern (Fig 6). This analysis did not predict which peptides would bind to MHC class II or which proteins could be cross-reactive to host proteins and impact the T cell repertoire. However, it is unlikely that there should be an inherent bias among the various patterns. Our pattern suggests *Bacteroides*-reactive T cells that respond to multiple species of bacteria may be common in the T cell repertoire.

## Discussion

Even though a large number of studies have characterized microbial changes associated with disease, no single bacterial species has been identified as causative of IBD or has been associated with the development of autoimmune diseases. On the other hand, T cell reactivity toward bacterial antigens, particularly from *Bacteroides* species, has been recognized as part of IBD pathogenesis.

We identified a CD4^+^ T cell hybridoma that recognized multiple *Bacteroides* species. We then characterized and defined the shared epitope that this T cell recognized. Mapping of the sequences obtained from AP-PCR against the *B*. *thetaiotaomicron* VPI 5482 genome showed that the transposon insertion knocked-down gene BT0900 of the wild type strain. This gene codes for a tetratricopetide repeat family containing protein (hypothetical protein), which contains the epitope sequence AKPFYEKARALK that stimulates the 52.13 TCR. Tetraticopeptide repeats have been associated to protein-protein interactions as chaperones, and with cell cycle, protein transport, transcription, and host-defense [20,21], among other functions. Even though this protein has not been characterized in *B*. *thetaiotaomicron*, it is of importance that analysis of mutant counts in chemostats and ex-germ-free mice colonized with a mixture of *B*. *thetaiotaomicron* transposon mutants [19] showed a decrease in the number of BT0900 mutants *in vivo* relative to *in vitro* cultures, thus implying a role for this gene in fitness and niche establishment. BT0900 is highly expressed *in vivo*, which may contribute to immunogenicity. Additionally, gene expression profiling of *B*. *thetaiotaomicron* has demonstrated that BT0900 is one of the top 2% of transcripts expressed both *in vivo* and *in vitro* [22]. We may speculate that T cell epitopes of gut microbes will be disproportionately derived from the top proteins expressed *in vivo*. However, additional studies of the T cell repertoire will be required to prove this hypothesis.

Our study represents the first time a T cell has been isolated from a *Bacteroides*-monoassociated gnotobiotic mouse. The T cell was then demonstrated to be specific for a colonizing-bacterial peptide without any *a priori* knowledge of the epitope. Despite the fact that *Bacteroides* have been studied for decades in both pathologic and symbiotic models in mice and as well as humans, this is the first T cell epitope that has been mapped to the peptide or protein level of specificity for this major group of gut microbes.

Our work presents a pipeline for the identification and characterization of bacterial antigenic epitopes recognized by T cells that are naturally primed *in vivo*. We demonstrated its efficacy by identifying the first T cell epitope within the genus *Bacteroides*. Results of this study provide tangible insight into the complex host T cell response to gut bacteria. This peptide has been predicted to be in 4 species of bacteria, yet other epitopes will certainly be shared by none or any number of species (Fig 5). This has serious implications for the treatment of gut-specific diseases like IBD. In the situation of fecal microbiota transplants (FMT) that are being tested to treat IBD, it is likely that one species of *Bacteroides* may be displaced by another during FMT because the majority of heathy microbiotas contain multiple *Bacteroides* species. Furthermore, it is important to note that when we included *E*. *coli* in our studies, a species from completely different Phyla (*Proteobacteria*), we still observe that there are peptides shared between *E*. *coli* and all *Bacteroides*. Thus, the possibility of TCR degeneracy (that is, the recognition of multiple epitopes by the same TCR) could not be excluded. Whether cross-reactivity results from minimal residue similarity, or whether is caused by cross-reactivity to other peptides is still possible. In any case, T cells cross-reactivity suggests that targeting single bacterial species may not necessarily reduce disease severity. However, epitope characterization among putative pathobionts will likely allow the development of strategies to treat IBD or autoimmune diseases by the antigen-specific suppression of the immune response to gut bacteria.

In summary, our manuscript presents a novel approach for the identification of gut bacteria antigenic epitopes. Its efficacy was proven by the finding of the first T cell-peptide epitope within the genus *Bacteroides*, one of the main groups of human gut bacteria. We recognize that the T cell we used in our studies was not a dominant member of the T cell repertoire, and we acknowledge that there might be other potential epitopes within the genus as we observed some cross-reactivity within the species. Blocking one bacterial epitope might not be the cure for IBD or an autoimmune disease. In any case, the etiology of these diseases is still unknown, but gut bacteria has been recognized as part of it. Thus, the search for bacterial epitopes capable of eliciting immune responses plays a major part in the development of preventive and corrective strategies to achieve health.

## Supporting Information

S1 Appendix The ARRIVE Guidelines Checklist. (DOCX)
