# Correction: Identification and Phylogeny of the First T Cell Epitope Identified from a Human Gut *Bacteroides* Species

**DOI:** 10.1371/journal.pone.0146630

**Published:** 2016-01-05

**Authors:** The PLOS ONE Staff

The third author’s name is misspelled. The correct name is: Yi-Ju Shen. The publisher apologizes for the error.
