# Supplementary material for: Identification and Phylogeny of the First T Cell Epitope Identified from a Human Gut Bacteroides Species
Source: PLoS One. 2015 Dec 4;10(12):e0144382. doi: 10.1371/journal.pone.0144382 (PMC4670158; doi:10.1371/journal.pone.0144382)

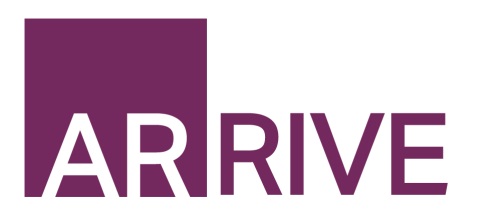


The ARRIVE Guidelines Checklist

Animal Research: Reporting In Vivo Experiments

Carol Kilkenny^1^, William J Browne^2^, Innes C Cuthill^3^, Michael Emerson^4^ and Douglas G Altman^5^

*^1^The National Centre for the Replacement, Refinement and Reduction of Animals in Research, London, UK, ^2^School of Veterinary Science, University of Bristol, Bristol, UK, ^3^School of Biological Sciences, University of Bristol, Bristol, UK, ^4^National Heart and Lung Institute, Imperial College London, UK, ^5^Centre for Statistics in Medicine, University of Oxford, Oxford, UK.*

|  | | ITEM | RECOMMENDATION | Section/ Paragraph |
| --- | --- | --- | --- | --- |
| 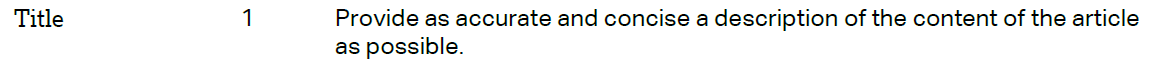 | | | Title |  |
| 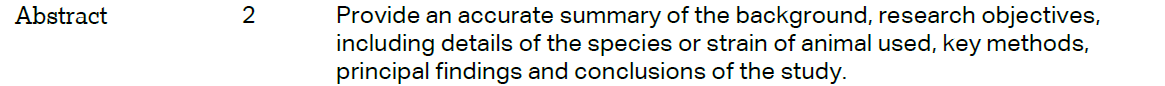 | | | Abstract |  |
| INTRODUCTION | | |  |  |
| 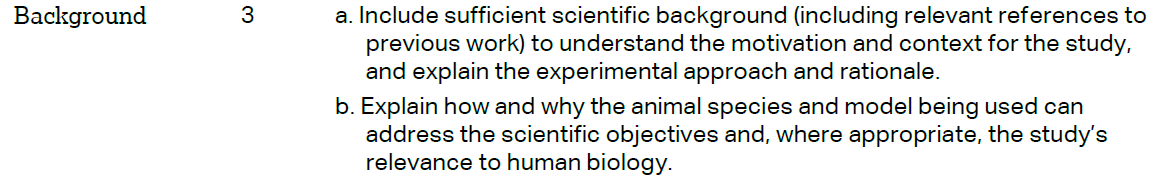 | | | Paragraphs 1-3 |  |
| 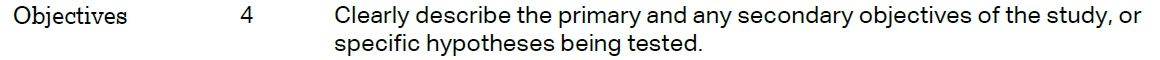 | | | Paragraphs 1-2 |  |
| METHODS | | |  |  |
| 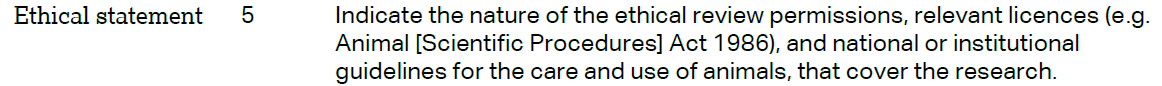 | | | Methods Paragraph 2 |  |
| 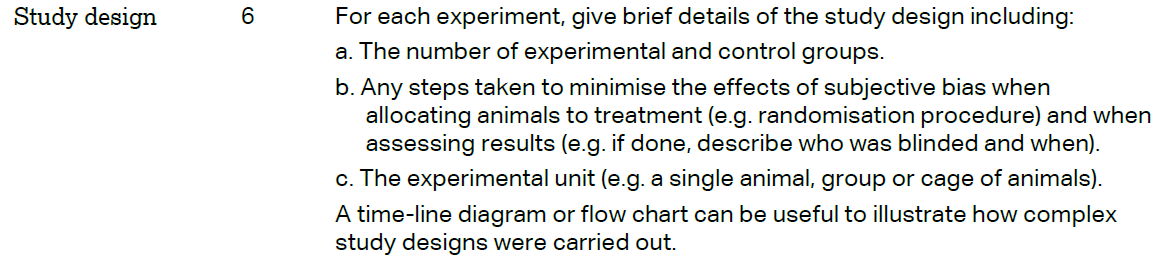 | | | Not applicable |  |
| 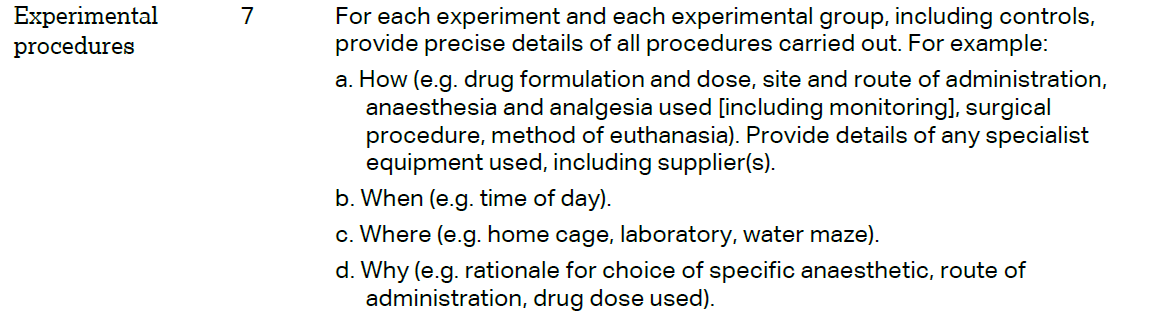 | | | Methods Paragraphs 2-3 |  |
| 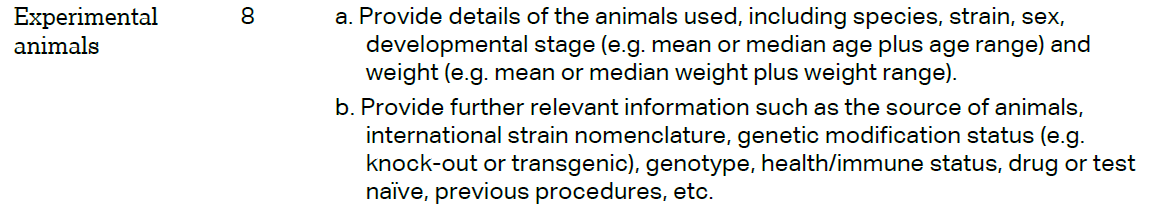 | | | Not applicable |  |

The ARRIVE guidelines. Originally published in *PLoS Biology*, June 2010^1^

| 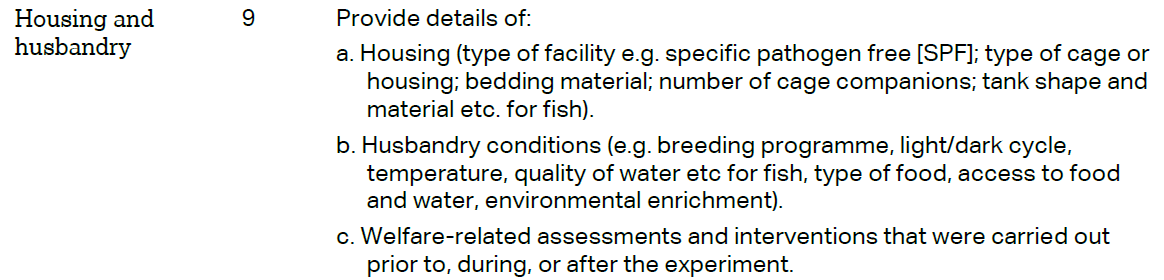 | Methods Paragraphs 1-2 |  |
| --- | --- | --- |
| 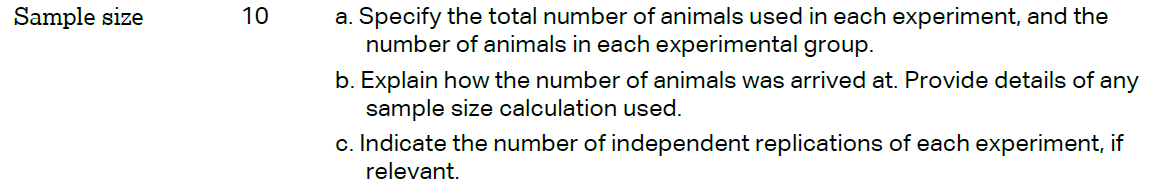 | No applicable |  |
| 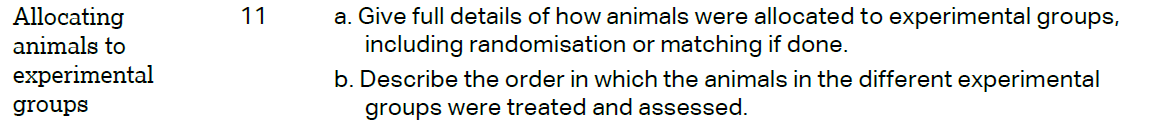 | Not applicable |  |
| 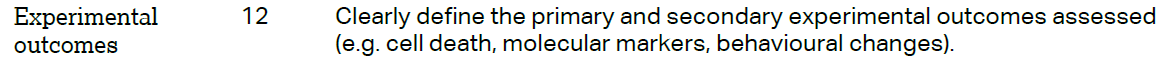 | Not applicable |  |
| 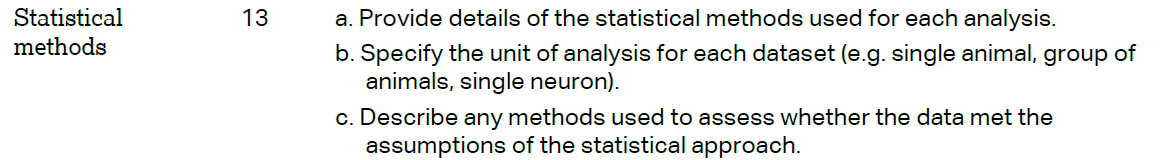 | Methods Paragraph 3 |  |
| RESULTS |  |  |
| 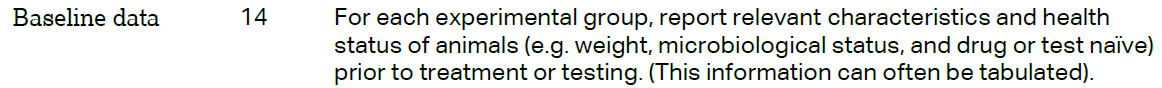 | Not applicable |  |
| 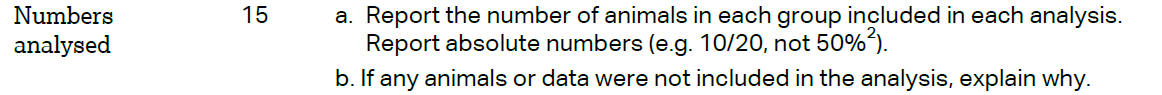 | Not applicable |  |
| 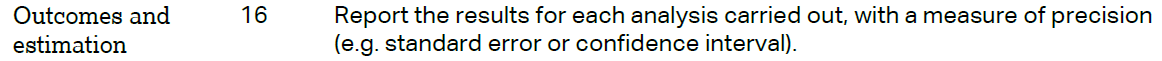 | Results Paragraphs 1-3 |  |
| 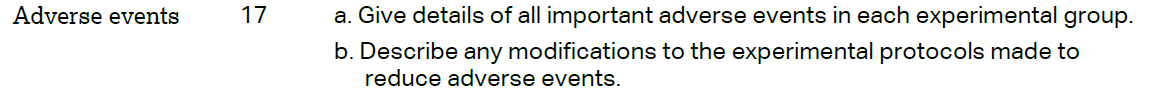 | Not applicable |  |
| DISCUSSION |  |  |
| 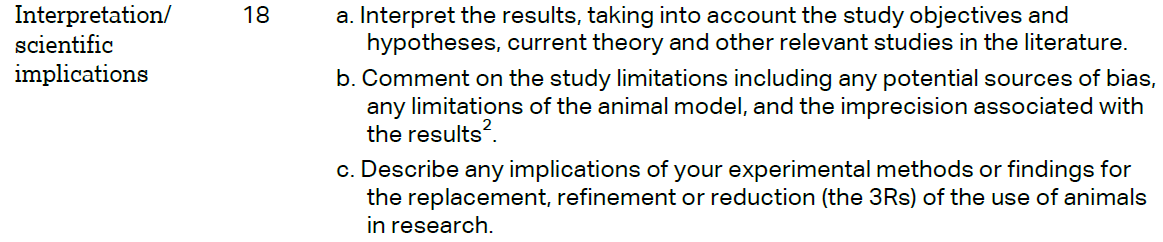 | Discussion Paragraphs 1-5 |  |
| 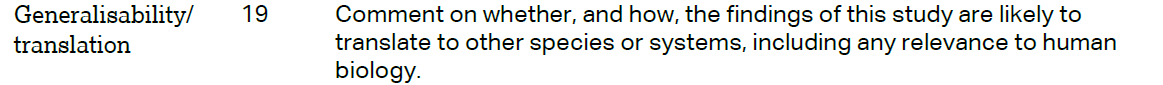 | Discussion Paragraph 3-5 |  |
| 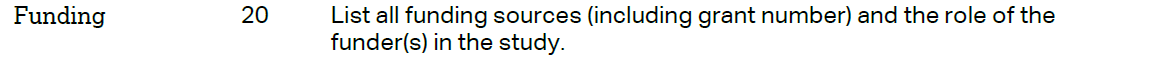 | | Online submission system |


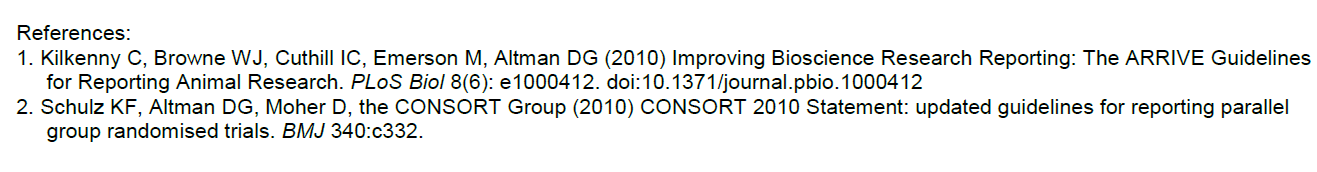

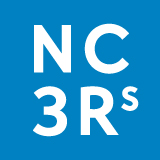

Supplement: S1 Appendix — (DOCX) [file pone.0144382.s001.docx]
